# Putative interaction of brush cells with bicarbonate secreting cells in the proximal corpus mucosa

**DOI:** 10.3389/fphys.2013.00182

**Published:** 2013-07-15

**Authors:** Julia Anna-Maria Eberle, Kai L. Müller-Roth, Patricia Widmayer, Vladimir Chubanov, Thomas Gudermann, Heinz Breer

**Affiliations:** ^1^Institute of Physiology, University of HohenheimStuttgart, Germany; ^2^Walther-Straub-Institute of Pharmacology and Toxicology, University of MunichMunich, Germany

**Keywords:** bicarbonate secretion, limiting ridge, brush cells, prostaglandins, PKD1L3

## Abstract

The gastric epithelium is protected from the highly acidic luminal content by alkaline mucus which is secreted from specialized epithelial cells. In the stomach of mice strong secretion of alkaline fluid was observed at the “gastric groove,” the border between corpus and fundus mucosa. Since this region is characterized by numerous brush cells it was proposed that these cells might secrete alkaline solution as suggested for brush cells in the bile duct. In fact, it was found that in this region multiple cells express elements which are relevant for the secretion of bicarbonate, including carbonic anhydrase (CAII), the cystic fibrosis transmembrane conductance regulator (CFTR) and the Na^+^/H^+^ exchanger (NHE1). However, this cell population was distinct from brush cells which express the TRP-channel TRPM5 and are considered as putative sensory cells. The location of both cell populations in close proximity implies the possibility for a paracrine interaction. This view was substantiated by the finding that brush cells express prostaglandin synthase-1 (COX-1) and the neighboring cells a specific receptor type for prostaglandins. The notion that brush cells may be able to sense a local acidification was supported by the observation that they express the channel PKD1L3 which contributes to the acid responsiveness of gustatory sensory cells. The results support the concept that brush cells may sense the luminal content and influence via prostaglandins the secretion of alkaline solution.

## Introduction

The extremely low pH value in the stomach lumen is important to ensure an adequate digestion of proteins and protection against ingested pathogens. Concurrently, despite the benefit of the abrasive acidic milieu, the integrity of the gastric mucosa has to be sustained. Protection of the mucosa against damaging effects of the gastric acid is mediated by a mucus layer which contains bicarbonate. The secretion of alkaline fluid by epithelial mucosal cells is elicited by various stimuli, including elevated luminal proton concentration (Garner and Hurst, 1980; Flemström and Kivilaakso, 1983; Takeuchi et al., 1986; Holm et al., 1998). In the stomach of rodents the intensity of alkaline secretion seems to differ significantly within various regions of the gastric mucosa. In a previous study it was observed that acidification of the gastric lumen elicited a rapid and significant rise of the pH in the proximal portion of the corpus close to the “limiting ridge,” whereas in the more distal parts of the corpus mucosa the pH remained strongly acidic (Akimori et al., 2011). Thus, an intense secretion of alkaline fluid seemed to occur at the corpus region adjacent to the fundus compartment. The alkalization at the corpus / fundus border may be important to protect the adjacent forestomach epithelium which lacks a protective mucus layer (Walsh, 2001). Thus, it seems conceivable that an enhanced secretion of alkaline fluid in the proximal parts of the corpus mucosa might create a pH-barrier between corpus and fundus compartment. In the stomach of rodents the boundary between the glandular corpus mucosa and keratinized fundus mucosa is the “limiting ridge,” a tissue fold over the “gastric groove” (Wattel and Geuze, 1978; Luciano and Reale, 1992). Beneath the tissue fold, a large number of brush cells are located and arranged in a palisade-like manner along the corpus/fundus border. Based on the recent observation that in the bile duct of rats brush cells express proteins, which are relevant for the generation and secretion of bicarbonate (Ogata, 2006), it has been speculated whether the brush cells at the “gastric groove” may also contribute to an enhanced alkaline secretion at the corpus/fundus border. Therefore, in this study attempts were made to evaluate whether distinct cells at the corpus/fundus transition have the capacity for secreting bicarbonate with a focus on a possible role of brush cells. We set out to identify and localize cells which express proteins involved in the generation and secretion of bicarbonate.

## Materials and methods

### Mice

Analyses were performed with wild type mouse strains C57/BL6J purchased from Charles River (Sulzfeld, Germany). Animals were fed with standard laboratory chow *ad libitum* and had free access to water. All experiments comply with the Principles of animal care, publication no. 85–23, revised 1985, of the National Institutes of Health and with the current laws of Germany. For tissue preparations animals were killed by cervical dislocation and subsequent decapitation or by inhalation of lethal doses of carbon dioxide delivered by a compressed gas cylinder.

### RNA isolation and cDNA synthesis

Total RNA was isolated from dissected tissue preparations of the stomach compartments with a Nucleo Spin RNA kit (Macherey-Nagel, Düren, Germany) according to the manufacturer's protocol. Therefor small tissue strips of comparable size were isolated out of the whole stomach. Corpus tissue, which is clearly distinguishable from other stomach compartments by means of its color, was prepared from the large curvature (Eberle et al., 2013). For the isolation of RNA from the corpus/fundus boundary a narrow strip of tissue was cut between the large and small curvature directly at the transition from corpus to fundus tissue where the “limiting ridge” proceeds. To ensure the complete removal of DNA, a DNase digestion (DNaseI, LifeTechnologies, Carlsbad, CA, USA) step was included. Purity of isolated RNA has been proven by measuring its ratio of the absorbance at 260 and 280 nm. Subsequently, 1.0 μg total RNA was reversely transcribed using oligo (dT) primers and SuperScript III Reverse Transcriptase (RT; Invitrogen, Carlsbad, CA, USA). RNA integrity of each sample was controlled by the amplification of the housekeeping gene for the ribosomal protein L8 (rpl8) with intron spanning primers to verify the DNA removal.

### Reverse transcriptase polymerase chain reaction (RT-PCR)

RT-PCR amplification was conducted by using normalized cDNA from different tissues of the stomach compartments. All genes were amplified using intron spanning primers to verify removal of genomic DNA. PCR amplifications were performed with the following primer combinations:

rpl8 forward, 5′-GTG CCT ACC ACA AGT ACA AGG C-3′; rpl8 reverse, 5′-CAG TTT TGG TTC CAC GCA GCC G-3′; CFTR forward, 5′-CTT GTG GAT GGG GGT TAT GTG CT-3′; CFTR reverse, 5′-CGA GGC TTG TGC TTG CTG GA-3′; CAII forward, 5′-TTG GAC TCA TGG ACA TAC CCT GGC-3′; CAII reverse, 5′-GCT ACA GAG AGG CGG TCA CAC TTG-3′; NHE1 forward, 5′-AGA ACA TCC ACC CCA AGG CTG-3′; NHE1 reverse, 5′-TCA TCT TCC TCC TCC TCC TCC G-3′; COX-1 forward, 5′-CTG ACA CAT GGA TAC TGG CTC TG-3′; COX-1 reverse, 5′-CGT GGG ATG CTC CTC CTT CAG C-3′; EP4 forward, 5′-TCT CTG GTG GTG CTC ATC TGC TC-3′; EP4 reverse, 5′-AGG TGG TGT CTG CTT GGG TCA-3′; PKD1L3 forward, 5′-CCT GAC CCT GCT GAT GAC TAC CG-3′; PKD1L3 reverse, 5′-GGA TTC CTAACA GAC TTG AGA GC-3′.

RT-PCR was carried out using High Fidelity PCR Enzyme Mix (Fermentas, St. Leon-Rot, Germany) and a Peltier PTC-200 thermo cycler (MJ Research). For amplification the following PCR cycling profiles were used with annealing temperatures adjusted to the used primer combinations and optimized numbers of amplification cycles, as specified in the following:

For rpl8:

One cycle: 4 min at 94°C, 25–28 cycles: 30 s at 94°C, 40 s at 65°C, 40 s at 72°C; and one cycle: 5 min at 72°C.

For CFTR:

One cycle: 4 min at 94°C; 20 cycles: 30 s at 94°C, 30 s at 66°C with −0.5°C per cycle, 40 s at 72°C; 20 cycles: 30 s at 94°C, 30 s at 56°C, 40 s at 72°C; and one cycle: 3 min at 72°C.

For CAII and COX-1:

One cycle: 4 min at 94°C; 20 cycles: 30 s at 94°C, 30 s at 68°C with −0.5°C per cycle, 40 s at 72°C; 7–12 cycles: 30 s at 94°C, 30 s at 58°C, 40 s at 72°C; and one cycle: 3 min at 72°C.

For NHE1:

One cycle: 4 min at 94°C; 20 cycles: 30 s at 94°C, 30 s at 63°C with −0.5°C per cycle, 40 s at 72°C; 25 cycles: 30 s at 94°C, 30 s at 53°C, 40 s at 72°C; and one cycle: 3 min at 72°C.

For EP4:

One cycle: 4 min at 94°C; 10 cycles: 30 s at 94°C, 30 s at 65°C with −0.5°C per cycle, 40 s at 72°C; 30 cycles: 30 s at 94°C, 30 s at 60°C, 40 s at 72°C; and one cycle: 3 min at 72°C.

For PKD1L3:

One cycle: 4 min at 94°C; 14 cycles: 30 s at 94°C, 30 s at 65°C with −0.5°C per cycle, 40 s at 72°C; 39 cycles: 30 s at 94°C, 30 s at 58°C, 40 s at 72°C; and one cycle: 3 min at 72°C.

PCR products were run on 1 or 1.5% agarose gels containing EtdBr. Amplification of a 204 bp fragment from mouse housekeeping control gene ribosomal protein l8 (rpl8) was used as control to confirm equal quality and quantity of the cDNA preparations.

### Tissue preparation

For *in situ* hybridization, the stomachs of adult mice were dissected in 1× phosphate-buffered saline (PBS: 0.85% NaCl, 1.4 mM KH2PO4, 8 mM Na2HPO4, pH 7.4), embedded in Leica OCT Cryocompound “tissue freezing medium” (Leica Microsystems, Bensheim, Germany) and quickly frozen on dry ice. Sections were cut on a CM3000 cryostat (Leica Microsystems, Bensheim, Germany) and adhered to Superfrost Plus microslides (Menzel Gläser, Braunschweig, Germany).

For immunohistochemistry, stomachs of adult mice were dissected in 1× PBS and fixed as described below.

For immunoreactivity to TRPM5, COX-1, PKD1L3 and CK18, tissue was fixed in 4% paraformaldehyde (in 150 mM phosphate buffer, pH 7.4) for 30 min to 2 h at 4°C.

After fixation the tissue was cryoprotected by incubation in 25% sucrose overnight at 4°C. Finally, the tissue was embedded in Tissue Freezing Medium and quickly frozen on dry ice or liquid nitrogen. Cryosections (4–8 μm) were generated using a CM3050S cryostat (Leica Microsystems, Bensheim, Germany) and adhered to Superfrost Plus microscope slides (Menzel Gläser, Braunschweig, Germany).

### *In situ* hybridization

The T7/SP6 RNA transcription system (Roche Diagnostics, Mannheim, Germany) was used, as recommended by the manufacturer, to generate digoxigenin-labeled antisense riboprobes from partial cDNA clones in pGem-T plasmids (subjected to sequence analysis in an ABI PRISM 310 Genetic Analyzer (Applied Biosystems, Foster City, Calif., USA)) encoding *Mus musculus* CFTR (Genbank accession number NM_021050.2, positions 4140–5351), CAII (Genbank accession number NM_009801.4, positions 344–1076), NHE1 (Genbank accession number NM_016981.2, positions 2037–3060), COX-1 (Genbank accession number NM_008969.3, positions 140–2363; positions 1112–2363), EP4 (Genbank accession number NM_008965.1, positions 1049–1496). The corresponding sense riboprobes were generated to serve as a negative control. Conditions for *in situ* hybridization were as described previously (Hass et al., 2010). Tissue sections were fixed in 4% paraformaldehyde / 0.1 M NaHCO3, pH 9.5 for 20–45 min at 4°C. For visualization of COX-1-mRNA tissue sections were hybridized with a mix of two antisense riboprobes. Sections were mounted in MOWIOL (10% polyvinyl-alcohol 4–88 (Sigma), 20% glycerol in 1× PBS).

### Immunohistochemistry

Cryosections (4–8 μm) were air-dried, rinsed in 1× PBS for 10 min at room temperature and blocked in 0.3% Triton X-100 in 1× PBS containing either 10% normal goat serum (NGS; Dianova, Hamburg, Germany) or 10% normal donkey serum (NDS; Dianova, Hamburg, Germany) for 30 min at room temperature. For immunostaining with COX-1, PKD1L3 and CK18 antibodies, cryosections underwent citrate-antigen-retrieval. Therefore, frozen sections were incubated in sodium citrate buffer (10 mM sodium citrate, 0.05% Tween 20, pH 6.0) for 45 min at 4°C. Afterwards sections were immersed in the same sodium citrate buffer for 5 or 10 min at 100°C. After three rinses for 5 min in 1× PBS, cryosections were blocked in 0.3% Triton X-100 in 1× PBS containing either 10% normal goat serum (NGS) or 10% normal donkey serum (NDS) for 30 min at room temperature. For single- and double-labeling experiments, primary antibodies were diluted in 0.3% Triton X-100 in 1× PBS containing either 10% NGS or 10% NDS. Antibodies were used in the following dilutions: rabbit anti-TRPM5 serum73 (purified antibody (AB-321) described in Kaske et al., 2007) 1:500 / 1:800; goat anti-COX-1 (sc-1754, Santa Cruz Biotechnology, Santa Cruz, CA, USA) 1:150; rabbit anti-PKD1L3 (ABIN571564, antibodies-online, Aachen, Germany) 1:100; mouse anti-cytokeratin18 (61028; Progen Biotechnik, Heidelberg, Germany) 1:80. Specificity of TRPM5 and COX-1 antibodies has been proven previously (Bezençon et al., 2008). Specificity of immunohistochemical stainings of the other antibodies were confirmed by *in situ* hybridization experiments. Blocked sections were incubated with the diluted primary antibodies overnight at 4°C. After washing in 1× PBS, the bound primary antibodies were visualized using appropriate secondary antibodies conjugated to Alexa 488 or Alexa 568 (Invitrogen, Karlsruhe, Germany, 1:500) diluted in 1× PBS with 0.3% Triton X-100 containing either 10% NGS or 10% NDS for 2 h at room temperature. After three rinses for 5 min in 1× PBS, the sections were counterstained with 4,6-diamidino-2-phenylindole (DAPI; l μg/mL, Sigma Aldrich, Schnelldorf, Germany) for 3 min at room temperature, rinsed with bidest and finally mounted in MOWIOL. No immunoreactivity could be observed when the primary antibodies were omitted.

### *In situ* hybridization combined with immunohistochemistry

To visualize TRPM5-immunoreactive cells on hybridized sections *in situ* hybridization was performed as described above. After one rinse for 10 min in 1× PBS, the sections were fixed for 3 min in 4% paraformaldehyde, rinsed three times for 5 min in 1× PBS and underwent standard immunohistochemical protocol.

### Microscopy and photography

Digital photographs of mouse stomach were taken with an Epson standard digital camera (Epson, Meerbusch, Germany). Immunohistochemical staining was documented by using a Zeiss Axiophot microscope (Carl Zeiss MicroImaging, Jena, Germany). Images were captured using a “Sensi-Cam” CCD-camera (PCOimaging, Kelheim, Germany). *In situ* hybridizations were photographed by using a Zeiss Axioskop 2 with an AxioCam MRc5 (Carl Zeiss MicroImaging, Göttingen, Germany). Images were adjusted for contrast in AxioVision LE Rel. 4.3 (Carl Zeiss MicroImaging, CityJena, Germany) and arranged in PowerPoint (Microsoft) and Adobe Photoshop (Adobe Systems, San Jose, CA, USA).

## Results

**Figure 1:**
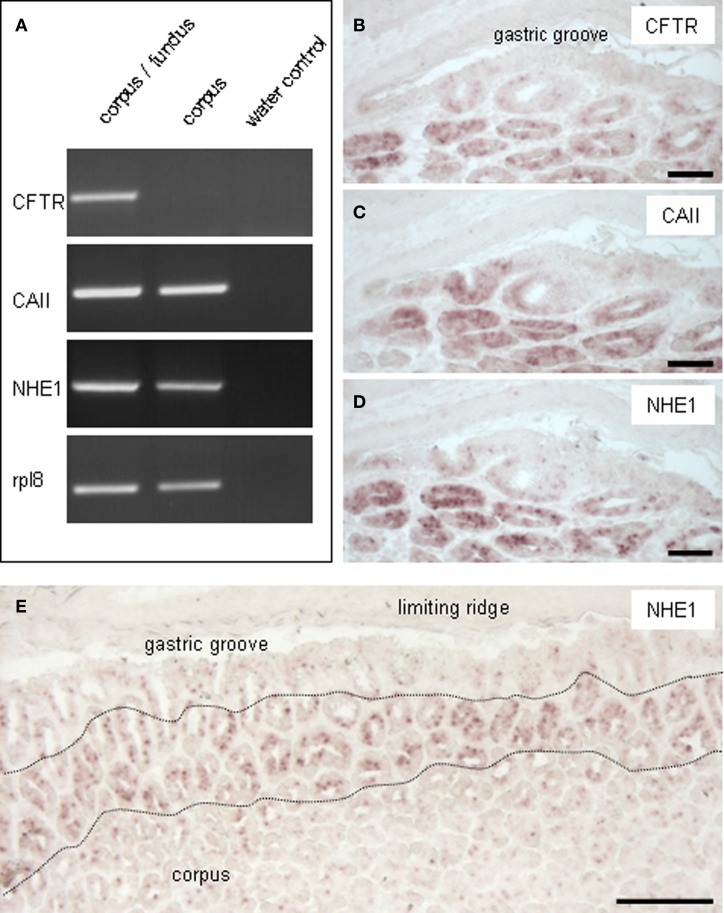
**Expression of CFTR, CAII and NHE1 in the corpus mucosa beneath the “limiting ridge.” (A)** Reverse transcription polymerase chain reaction (RT-PCR) experiments were performed with primer pairs specific for CFTR (368 bp), CAII (276 bp) and NHE1 (457 bp). Normalized cDNA from the corpus/fundus transition zone and the corpus, respectively, was analyzed and amplicons of the expected size were obtained. For CFTR the strongest band was amplified for the corpus/fundus transition zone. Water controls without template showed no amplicon. Amplification of the mouse housekeeping control gene ribosomal protein l8 (rpl8) was used as control to confirm equal quality and quantity of the cDNA preparations. **(B–D)** *In situ* hybridization experiments with antisense riboprobes for CFTR, CAII and NHE1 were performed on consecutive sections of the corpus mucosa. For all three analyzed elements a similar staining pattern was observed. The strongest signals were visible in cross sectioned invaginations of the most apical corporal mucosa, adjacent to the “gastric groove.” Few hybridization signals were visualized in the corpus epithelium forming the distal wall of the “gastric groove.” **(E)** *In situ* hybridization with an antisense probe for NHE1 showed the distribution of NHE1-expressing cells. Tissue sections which contained the band-like arrangement of the “limiting ridge” displayed strong signals in the corporal mucosa, visible in a band-like manner (denoted by the *black broken line*) parallel to the “limiting ridge.” The mucosal region facing the “gastric groove,” and the more distally located mucosal regions showed less and weaker signals. *Scale bars:* **(B–D)** = 50 μm, **(E)** = 100 μm.

In a previous study it has been demonstrated that upon injection of tetragastrin, which is supposed to elicit acidification of the luminal content, a band with an alkaline pH occurred in the most proximal region of the corpus mucosa, whereas the more distal parts of the gastric mucosa were highly acidic (Akimori et al., 2011). Based on this observation we set out to explore whether cells at the corpus/fundus border are particularly suited to secret bicarbonate. The capability of specialized cells to efficiently secret bicarbonate is based on distinct molecular elements, most notably the carbonic anhydrase (CAII), the cystic fibrosis transmembrane conductance regulator (CFTR) and the Na^+^/H^+^ exchanger 1 (NHE1) (Jacob et al., 2000; Steward et al., 2005; Ishiguro et al., 2009). Whether the three functional elements for secreting alkaline fluid are expressed in the gastric mucosa at the corpus/fundus boundary was analyzed by RT-PCR experiments and by *in situ* hybridization studies. Using cDNA prepared from tissue samples of the corpus/fundus region and from the more distal corpus region, respectively, RT-PCR analyses were conducted on at least three animals for each gene with specific primers and resulted in amplicons of the expected size (Figure 1A). For CFTR a strong band was obtained for the corpus/fundus boundary region, whereas for CAII and NHE1 amplicons of comparable size for both mucosal regions were received. For each RT-PCR analysis amplification of the mouse housekeeping control gene ribosomal protein l8 (rpl8) was used as control to confirm equal quality and quantity of the cDNA preparations, as exemplarily shown for Figure 1A. RT-PCR experiments shown in Figures 1, 5, 6 were conducted with the same cDNA, hence the representative PCR result for rpl8 is the same for the respective figures. In order to determine the location of cells which express the relevant elements, consecutive sections of the corpus mucosa close to the corpus/fundus boundary were hybridized with respective riboprobes. The results depicted in Figures 1B–D indicate a strong staining of cross sectioned annuli located in the proximal corpus mucosa, in the proximity to the “gastric groove.” The strikingly similar staining pattern for all three elements on consecutive sections suggests that they are probably co-expressed in epithelial cells lining the annuli at the proximal corpus. *In situ* hybridization analyses for the respective elements were conducted on at least three animals. Hybridization with the corresponding sense riboprobes didn't show signals.

To unravel the distribution of labeled cells along the corpus/fundus boundary, tissue sections were analyzed which comprised the prolonged arrangement of the tissue fold, the so-called “limiting ridge” (Figure 1E). As shown exemplarily for NHE1, labeled cells of the epithelium lining the cross sectioned annuli extended in a band-like manner parallel to the “limiting ridge.” The proximal small strip, which forms the distal wall of the “gastric groove” as well as the more distally located corpus regions showed considerably less and much weaker labeling.

**Figure 2:**
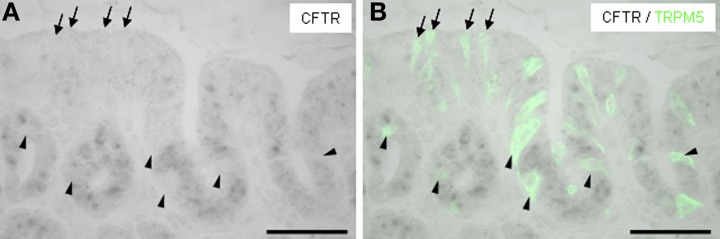
**Pattern of CFTR hybridization signals and TRPM5-immunostaining in the corpus mucosa. (A)** *In situ* hybridization on sections of the corporal mucosa with an antisense riboprobe for CFTR. Intense labeling was mainly observed at the bottom of invaginations opening into the “gastric groove” and in cross sectioned annuli adjacent to the “gastric groove.” *Arrowheads* and *arrows*, respectively, depict the regions which were devoid of hybridization signals but immunoreactive for TRPM5 in **(B)**. **(B)** *In situ* hybridization for CFTR combined with immunohistochemical staining for TRPM5 (*green*) revealed different expression patterns. Several TRPM5-positive cells (designated by *arrows*) were visible in the corpus mucosa forming the distal wall of the “gastric groove” where only few and weak hybridization signals were observed. Within the invaginations opening into the “gastric groove” several TRPM5-immunoreactive cells (*arrowheads)* were localized in direct proximity to cells strongly stained by the riboprobe for CFTR. Less TRPM5-positive cells (also indicated by *arrowheads*) were visible in cross sectioned annuli next to cells with intense hybridization signals. *Scale bars:* **(A,B)** = 50 μm.

**Figure 3:**
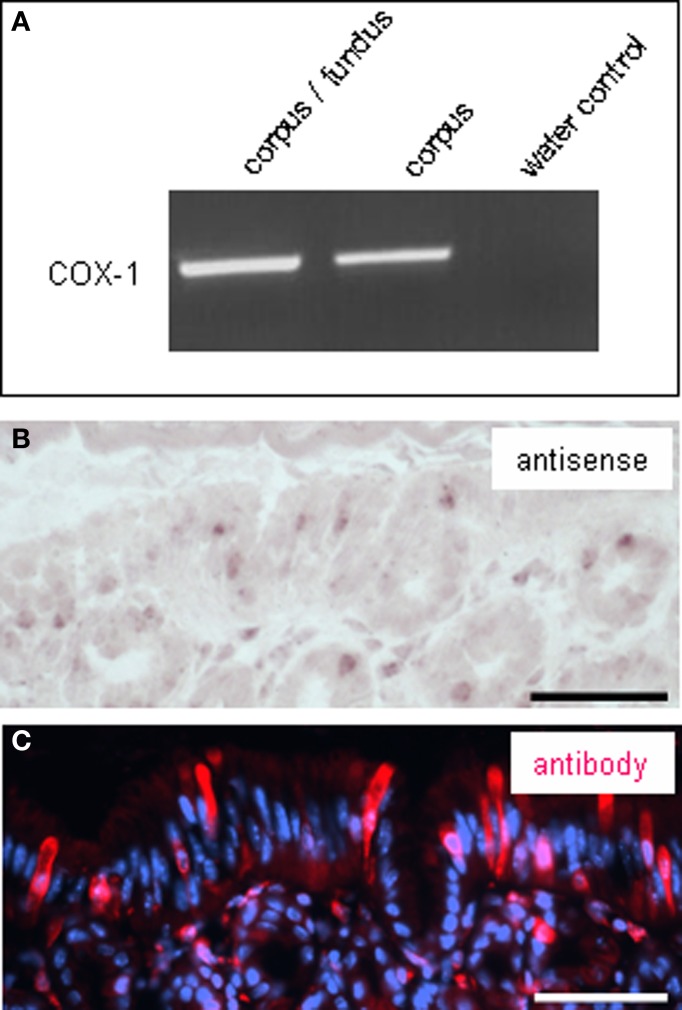
**Expression of cyclooxygenase-1 (COX-1) in the corpus mucosa. (A)** RT-PCR experiments with primer pairs specific for COX-1 (690 bp). Amplicon of the expected size from normalized cDNA of tissue from the corpus/fundus transition zone and corpus was obtained. **(B)** *In situ* hybridization experiments with an antisense riboprobe for COX-1. Several signals were visible in the epithelial corpus mucosa forming the distal wall of the “gastric groove” and in cross sectioned annuli adjacent to the “gastric groove.” **(C)** Immunohistochemical stainings with a COX-1-antibody. Pattern of COX-1-immunostaining (*red*) is similar to COX-1-hybridization pattern shown in **(B)**. The mucosal region forming the “gastric groove” and the adjacent corpus mucosa showed immunoreactive cells. Sections were counterstained with DAPI (*blue*). *Scale bars:* **(B,C)** = 50 μm.

**Figure 4:**
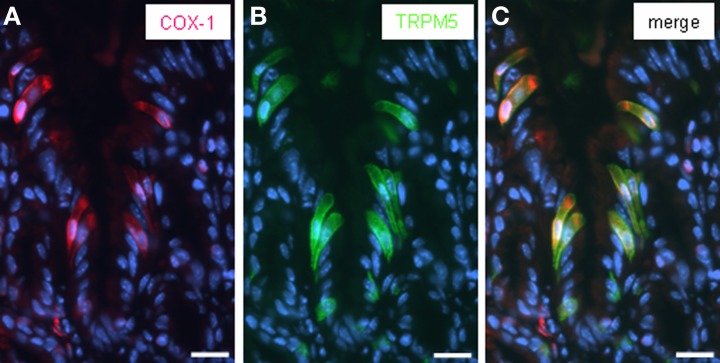
**Immunohistochemical visualization of COX-1 in TRPM5-expressing cells. (A)** Immunostaining of tissue sections of the corporal mucosa region underneath the “limiting ridge” with an antibody against COX-1. Within the longitudinally sectioned invagination which opened into the “gastric groove” several COX-1-expressing cells (*red*) were stained. **(B)** The TRPM5-antibody revealed a staining pattern *(green*) reminiscent of that in **(A)**. **(C)** Overlay of **(A)** and **(B)** clearly demonstrated coexpression of COX-1 and TRPM5 in a subset of cells within the invagination. Sections were counterstained with DAPI (*blue*). *Scale bars*: **(A–C)** = 20 μm.

**Figure 5:**
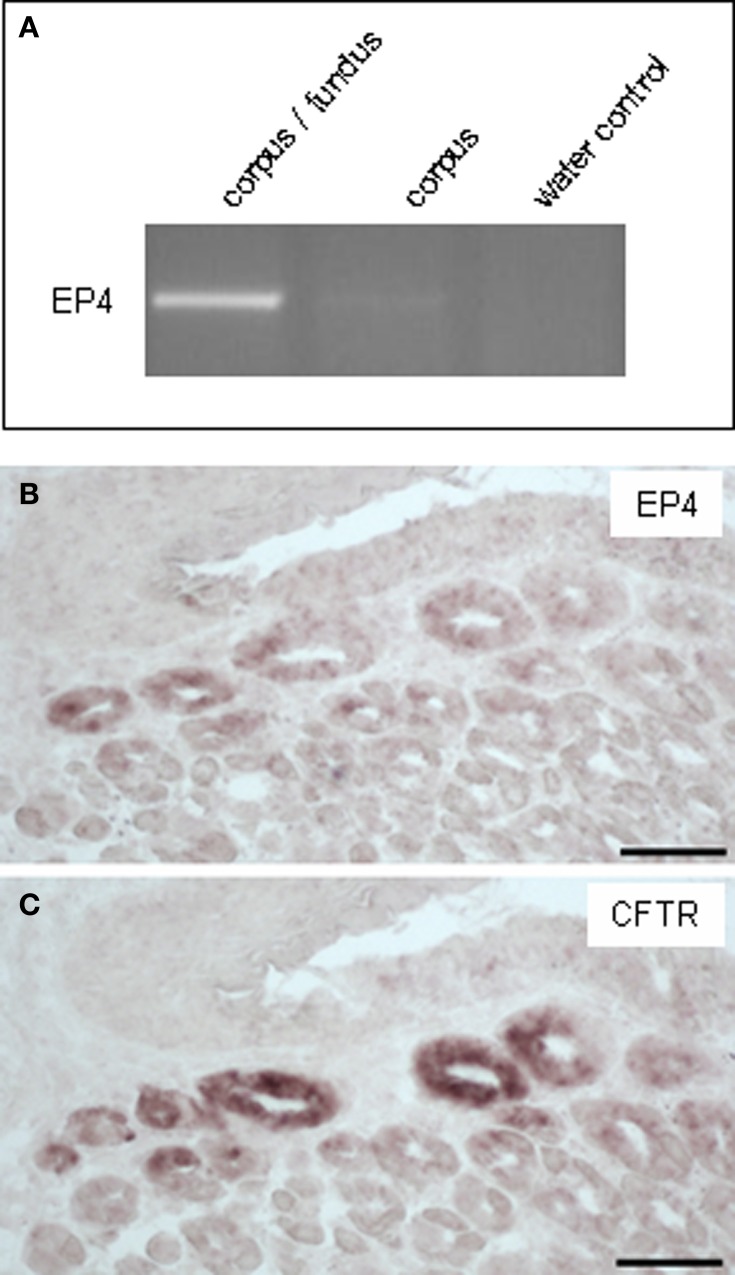
**Expression of the prostaglandin E2 receptor subtype EP4 in the apical corpus mucosa adjacent to the “gastric groove.” (A)** RT-PCR approaches were conducted with primer pairs specific for EP4 (448 bp). Normalized cDNA of gastric tissue from the corpus/fundus boundary and corpus was analyzed; rather a weak band for EP4 was obtained for corpus cDNA, a stronger band was amplified for cDNA of the corpus/fundus transition zone. **(B)** *In situ* hybridization analyses with an antisense riboprobe for EP4; strong staining in cross sectioned annuli of the apical corpus mucosa close to the “gastric groove.” In more distally located annuli no signals were observed. **(C)** Hybridization of a consecutive section with an antisense riboprobe for CFTR; the staining pattern is reminiscent of that in **(B)**. *Scale bars*: **(A,B)** = 50 μm.

It has recently been suggested that rats' brush cells in the bile duct may be important for the secretion of bicarbonate; this notion was based on the observation that brush cells within bile duct epithelium express relevant proteins, such as CAII, CFTR and NHE1 (Ogata, 2006). Within the gastric mucosa a particularly high number of brush cells are located close to the “gastric groove” (Akimori et al., 2011; Eberle et al., 2013); since the alkalization was observed in this region it was speculated that the brush cells at the “gastric groove” may be involved in the secretion of alkaline fluid (Akimori et al., 2011). Therefore, brush cells were analyzed for the expression of elements relevant for bicarbonate secretion, using TRPM5 (Hofmann et al., 2003) as marker for most brush cells at the “gastric groove” (Eberle et al., 2013) and CFTR as a marker for HCO^−^_3_-secreting cells. Using a technique which allows to simultaneously visualize mRNA by *in situ* hybridization and protein by immunohistochemistry, we found strong hybridization signals for CFTR in the most proximal invaginations of the corpus mucosa (Figure 2A); both, cross-sectioned invaginations as well longitudinal-sectioned invaginations, are visible in Figure 2. An overlay with the immunohistochemical labeling for TRPM5 (Figure 2B) revealed that all immunoreactive cells were devoid of hybridization signals. Neither the TRPM5-positive cells in invaginations (marked by *arrowheads* in Figure 2) nor at the “gastric groove” (marked by *arrows* in Figure 2) showed any indication for CFTR expression. Also for CAII and NHE1, respectively, hybridization signals didn't overlap with immunohistochemical stainings for TRPM5. These data indicate that cells expressing elements essential for HCO^−^_3_ secretion and the TRPM5-positive cells represent two distinct populations and that in the proximal corpus mucosa apparently brush cells are not the source for secreted bicarbonate. However, the observation that TRPM5-positive cells were frequently located in close proximity to CFTR-stained cells might be of functional relevance, especially in view of the previous findings that in other tissues secretion of bicarbonate is strongly affected by neighboring cells using prostaglandin as paracrine signal (Flemström et al., 1982; Takeuchi et al., 1999; Sugamoto et al., 2001; Akiba and Kaunitz, 2009). If such a scenario was also realized at the “gastric groove,” the brush cells need to comprise the enzyme prostaglandin-endoperoxide synthase, also called cyclooxygenase (COX) [reviewed by Kim (2011)]. Interestingly, in a recent study it has been demonstrated for the murine duodenum that TRPM5-expressing cells are immunoreactive for this enzyme (Bezençon et al., 2008). To analyze whether brush cells which are positioned in close proximity to CFTR-expressing cells may have the capacity to synthesize prostaglandins, RT-PCR analyses were performed with tissue samples from the different corpus regions resulting in amplicons which indicate the expression of COX-1 for both regions; a slightly stronger band was obtained for the corpus/fundus transition zone (Figure 3A). Amplification of the mouse housekeeping control gene ribosomal protein l8 (rpl8) was used as control to confirm equal quality and quantity of the cDNA preparations, as performed and shown for Figure 1A. In subsequent *in situ* hybridization experiments a variety of labeled cells were visible located adjacent to the “gastric groove” (Figure 3B). Hybridization with the sense riboprobe for COX-1 yielded no hybridization signals. The indication for COX-1 expressing cells in this region was confirmed by immunohistochemical experiments using a COX-1-antibody (Figure 3C). Both *in situ* hybridization and immunohistochemical approaches for COX-1 has been conducted on at least three animals. To approach the question whether the COX-1-expressing cells might be brush cells, double-labeling experiments with COX-1 and TRPM5-antibodies were performed. The results shown in Figure 4C indicate that the COX-1 expressing cells (Figure 4A) also expressed TRPM5 (Figure 4B). Thus, brush cells at the proximal corpus mucosa appear to be capable of synthesizing prostaglandins. Prostaglandins can mediate their effects via G-protein-coupled receptors including the four subtypes EP1-EP4 (reviewed by Sugimoto and Narumiya, 2007). Thus, a prerequisite for a prostaglandin-mediated regulation of bicarbonate secretion would be the expression of an appropriate receptor in the secretory cells. There is some evidence indicating that the influence of prostaglandins on bicarbonate secretion in the duodenum of rats is mediated via the receptor subtype EP4 (Aoi et al., 2004). RT-PCR analyses with normalized cDNA from the corpus/fundus boundary region and from more distally located corpus regions, respectively, resulted in a stronger band for the corpus/fundus transition (Figure 5A). *In situ* hybridization experiments with antisense riboprobes for EP4 led to hybridization signals in the most proximal invaginations adjacent to the “gastric groove” (Figure 5B), whereas the sense riboprobe showed no signals. This staining pattern was reminiscent to that for CFTR, CAII and NHE1 (Figures 1B–D) and was observable repeatedly on three analyzed animals. To approach the question whether the prostaglandin receptor subtype EP4 may in fact be expressed in cells with the capacity for bicarbonate secretion, consecutive tissue sections were hybridized with riboprobes for EP4 and CFTR, respectively. The results depicted in Figures 5B,C indicate a similar staining pattern for both factors. Thus, it seems possible that the capacity of cells to synthesize and secrete bicarbonate might be affected by prostaglandins released from brush cells.

**Figure 6:**
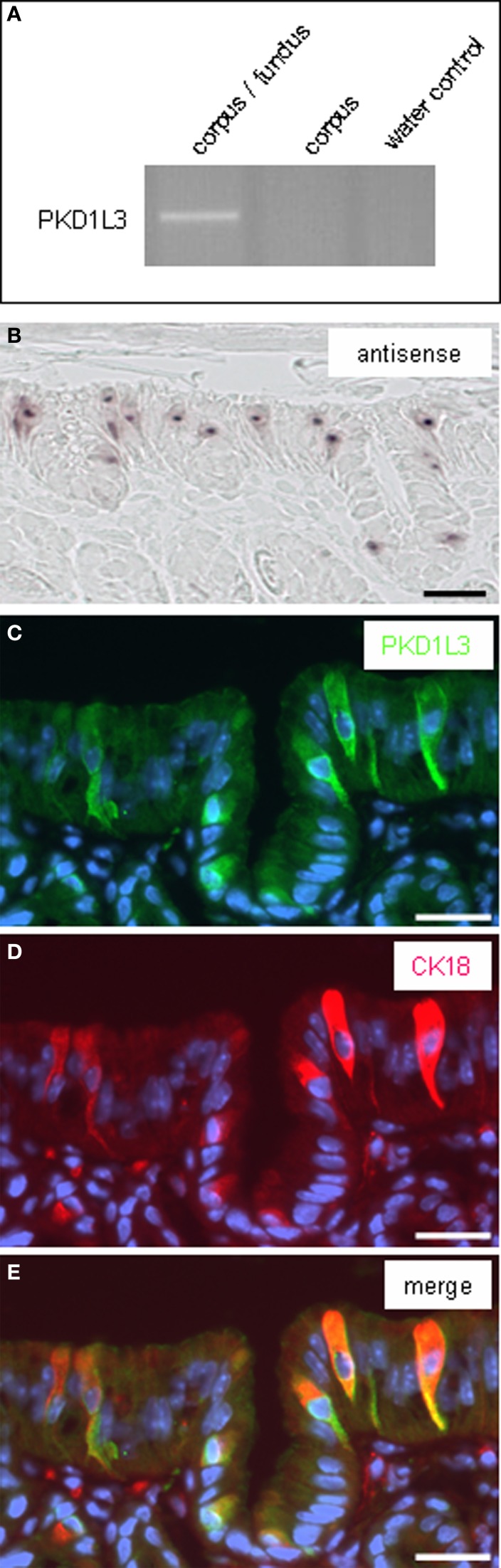
**Expression of PKD1L3 in CK18-positive brush cells of the corpus mucosa beneath the “limiting ridge.” (A)** RT-PCR experiments with primer pairs specific for PKD1L3 (460 bp). A distinct band of the expected molecular size was amplified for PKD1L3 from cDNA of the corpus/fundus boundary and corpus mucosa. Water controls lacking template showed no amplicon. **(B)** *In situ* hybridization with an antisense riboprobe for PKD1L3. Several hybridization signals got visible in the apical corpus mucosa underneath the “limiting ridge.” **(C)** Immunostaining with a PKD1L3-antibody labeled cells (*green*) in the apical corpus mucosa at the “gastric groove” and within the invagination. **(D)** The CK18-antibody stained cells (*red*) in the same region. **(E)** Overlay of the two images **(C,D)** clearly demonstrates that the cells were stained by both the PKD1L3- and CK18-antibody. Sections were counterstained with DAPI (*blue*). *Scale bars*: **(B–E)** = 20 μm.

Previous work has demonstrated that besides neuro-humoral factors also an acidic luminal content affects the secretion of bicarbonate in the duodenum and this effect is accompanied with an increase of the mucosal PGE_2_ content (Sugamoto et al., 2001; Takeuchi et al., 2002). Thus, one might assume that acidification of the gastric lumen can lead to a release of prostaglandins. This would imply that brush cells are capable to sense the luminal proton concentration. For taste cells it has been proposed that the potential acid sensor PKD1L3 may contribute to sense a low pH (Ishimaru et al., 2006; Kawaguchi et al., 2010). RT-PCR experiments with cDNA from the corpus/fundus transition zone and from corpus mucosa resulted in amplicons for PKD1L3 (Figure 6A). *In situ* hybridization with antisense riboprobes for PKD1L3 resulted in several stained cells in the apical corpus mucosa underneath the “limiting ridge” (Figure 6B), whereas the sense riboprobe showed no signals. Immunohistochemical approaches with a PKD1L3-antibody led to the visualization of several immunoreactive cells at the “gastric groove” (Figure 6C). Histochemical stainings with the respective riborpobes and antibody were conducted on at least three animals. Application of an antibody against CK18, a marker for brush cells, gave a similar labeling pattern (Figure 6D); an overlay demonstrated the overlap of immunoreactivity for PKD1L3 and CK18 (Figure 6E). These results indicate that brush cells in the proximal corpus mucosa apparently express the channel protein PKD1L3 and might be capable of sensing changes in the proton concentration of the luminal content.

## Discussion

Despite the remarkable progress in elucidating the molecular phenotype of brush cells, the functional role of these cells in the gastric mucosa still remains elusive. This is especially true for brush cells which are arranged in a palisade-like fashion at the “gastric groove,” the transition zone between corpus and fundus region of the rodent stomach. Based on the observation that a tetragastrin-induced acidification of the corpus lumen was accompanied by an alkalization of a small area at the corpus/fundus border, it was speculated that brush cells at the “gastric groove” may be the source for the alkaline fluid (Akimori et al., 2011). In this study, we have scrutinized this hypothesis by identifying cells in this region which have the molecular capacity to synthesize and secrete bicarbonate. Generally, such cells are characterized by typical functional elements, including carbonic anhydrase II (CAII), cystic fibrosis transmembrane conductance regulator (CFTR) and the Na^+^/H^+^ exchanger 1 (NHE1) (Flemström and Isenberg, 2001; Konturek et al., 2004; Allen and Flemström, 2005; Steward et al., 2005; Ogata, 2006; Sindić et al., 2010). Using histochemical approaches cells which express these elements were visualized and identified in the proximal corpus mucosa region, close to the “gastric groove.” The results indicate that the candidate cells for producing alkaline solution in this region are not the brush cells as previously proposed (Akimori et al., 2011) but rather specialized epithelial cells located within invaginations at the proximal corpus mucosa. Interestingly, these cells are frequently located in close proximity to brush cells. Due to their molecular phenotype brush cells are considered to have sensory capacity (Höfer et al., 1996; Hass et al., 2007, 2010; Eberle et al., 2013). Thus, it seems possible that some of these potentially sensory cells are specialized to sense changes in the luminal pH milieu and convey information onto adjacent HCO^−^_3_-producing cells. This notion is supported by the finding that CK18-positive cells at the “gastric groove” express PKD1L3 which is a subunit of a potential acid-sensing channel complex, which renders the type III taste cells responsive to low pH (Huang et al., 2006; Ishimaru et al., 2006; Kawaguchi et al., 2010). Thus, it seems possible that PKD1L3-expressing brush cells at the “gastric groove” may be capable of sensing the increase of proton concentration which occurs upon gastric acid secretion. Furthermore, the cells may be able to convey this information onto the HCO^−^_3_-producing cells in a paracrine fashion. In this context it was an important finding that brush cells at the “gastric groove” express the enzyme COX-1 which catalyzes the synthesis of prostaglandin. COX-1 expression has also been found for TRPM5 cells in the duodenum (Bezençon et al., 2008). Several studies have demonstrated that in the duodenum prostaglandins (PGs), especially PGE_2_, strongly affect the secretion of bicarbonate (Flemström et al., 1982; Takeuchi et al., 1986; Aly and Flemström, 1988). Similarly, in the pancreas prostaglandins also stimulate the secretion of bicarbonate (Saad et al., 1987). A similar role of prostaglandins has also been proposed for the gastric mucosa, since inhibition of COX-1 has led to a significantly reduced alkali secretion (Baumgartner et al., 2002). Prostaglandins serve as signal molecule controlling secretory activity also in other tissues; e.g., in the kidney, they act as transmitters from macula densa cells to juxtaglomerular cells modulating the exocytosis of renin (Jensen et al., 1996; Friis et al., 2005; Kim et al., 2007). Prostaglandins are fatty acid derivatives and known for their local action on adjacent target cells (Kauffman et al., 1980; Takeuchi et al., 2011). Thus, the close association of brush cells and CFTR-positive cells (Figure 2B) may indicate a paracrine signaling between the respective cell types by means of prostaglandins. This concept was further substantiated by the finding that within annuli of the most proximal corpus region, close to the “gastric groove,” cells are localized which express the prostaglandin receptor subtype EP4; this receptor is also involved in the PG-induced HCO^−^_3_-secretion within the duodenum (Aoi et al., 2004; Aihara et al., 2007).

The finding that brush cells at the “gastric groove” are probably not the source for secreted alkaline solution but rather may contribute to activate the relevant cells may point to a functional interplay between sensing brush cells and bicarbonate secreting mucosal cells. The position of this cellular assembly at the junction between corpus and fundus is probably of particular relevance since it would contribute to generate a pH barrier between the stomach compartments and prevent an acidification of stored food in the proximal part of the stomach. This conception is supported by the observation that higher pH values were measured in the fundus lumen compared to the lumen of the corpus (Smith, 1965; Walsh, 2001). The more neutral pH in the fundus region may be of particular importance, since in mice and rats, other than in humans, rabbits and dogs, this proximal compartment of the stomach consists of non-glandular tissue, which lacks a protective mucus layer (Walsh, 2001). Thus, more neutral conditions may be important to maintain the integrity of the epithelium. Along the “limiting ridge,” which encircles the whole circumference of the stomach and separates fundus from corpus (Wattel and Geuze, 1978; Luciano and Reale, 1992), both, the brush cells at the “gastric groove” (Eberle et al., 2013) and cells expressing elements for bicarbonate secretion (Figure 1E), are arranged in a band-like manner; thus, an enhanced secretion of bicarbonate all along the border between glandular and non-glandular tissue may contribute to protect the fundic epithelium from an abrasive acid milieu. More distally, the corpus mucosa is reliant on an acidic milieu of the gastric juice, necessary to ensure an effective digestion of the diet.

Thus, the present findings suggest that cells at the “gastric groove” are involved in maintaining different pH milieus in the proximal and in the distal murine stomach, thus contributing to the integrity of the gastric mucosa and adequate conditions for digestive processes in the stomach.

### Conflict of interest statement

The authors declare that the research was conducted in the absence of any commercial or financial relationships that could be construed as a potential conflict of interest.

## Abbreviations

CFTR: cystic fibrosis transmembrane conductance regulator
CA: carbonic anhydrase
NHE: Na^+^/H^+^ exchanger
COX: cyclo-oxygenase, EP4, prostaglandin E2 receptor subtype EP4
PKD1L3: polycystic kidney disease 1 like 3
CK18: cytokeratin 18
TRPM5: transient receptor potential cation channel, subfamily M, member 5.
