# Supplementary material for: Putative interaction of brush cells with bicarbonate secreting cells in the proximal corpus mucosa
Source: Front Physiol. 2013 Jul 15;4:182. doi: 10.3389/fphys.2013.00182 (PMC3711009; doi:10.3389/fphys.2013.00182)

attached figure 1

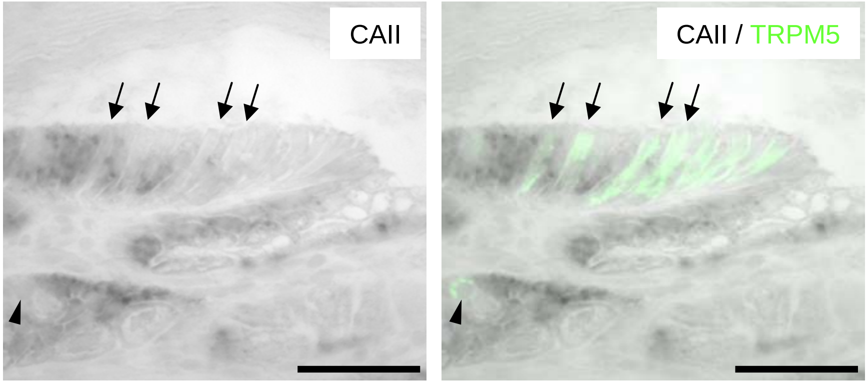

attached figure 2

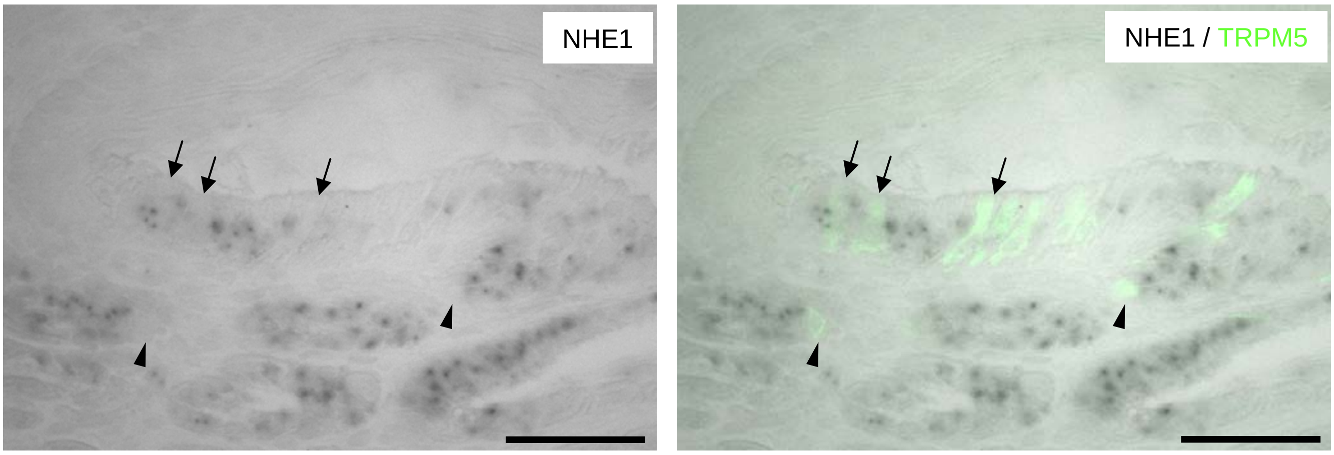

attached figure 3

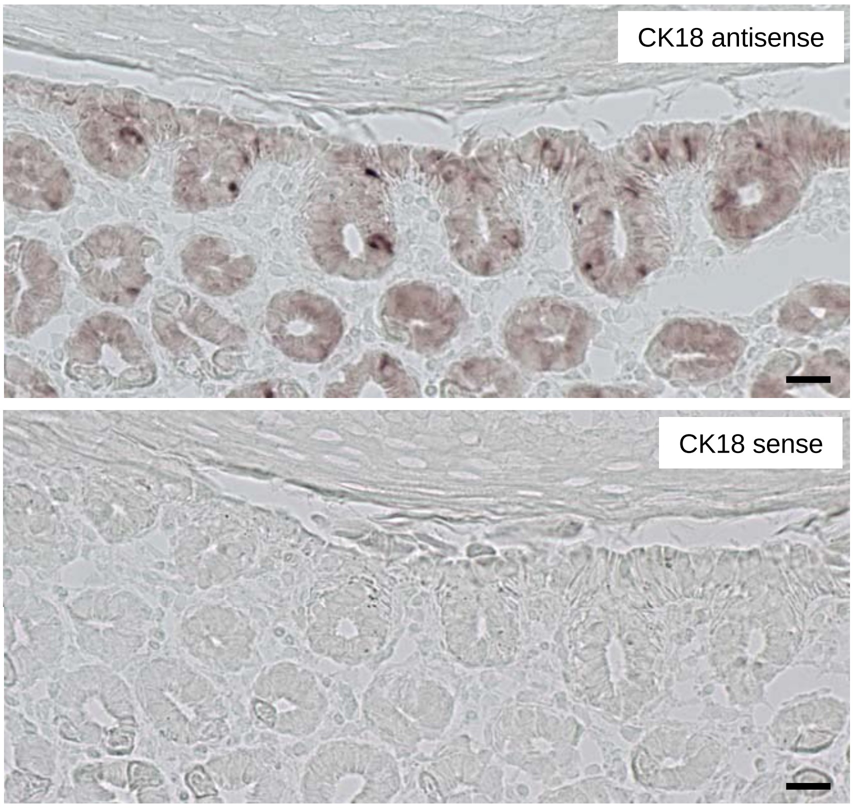

attached figure 4

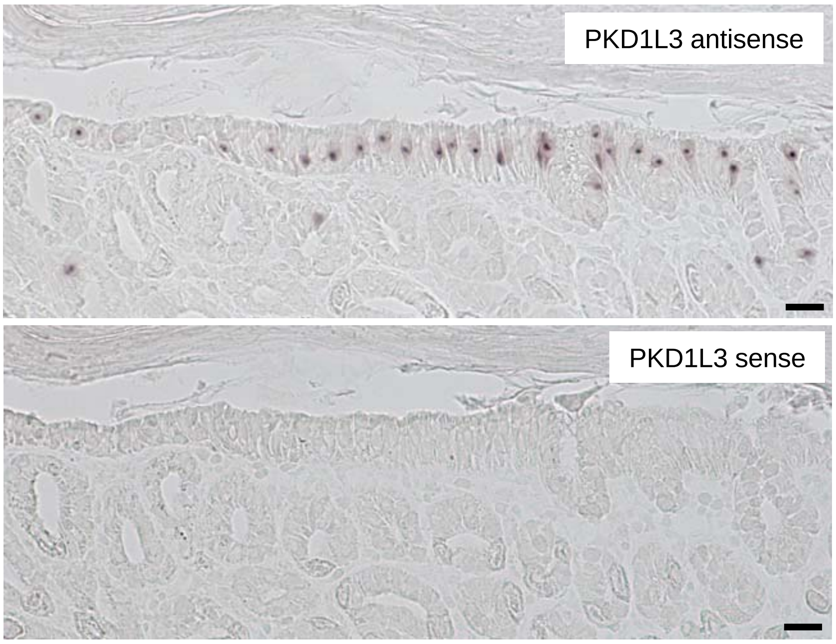

attached figure 5

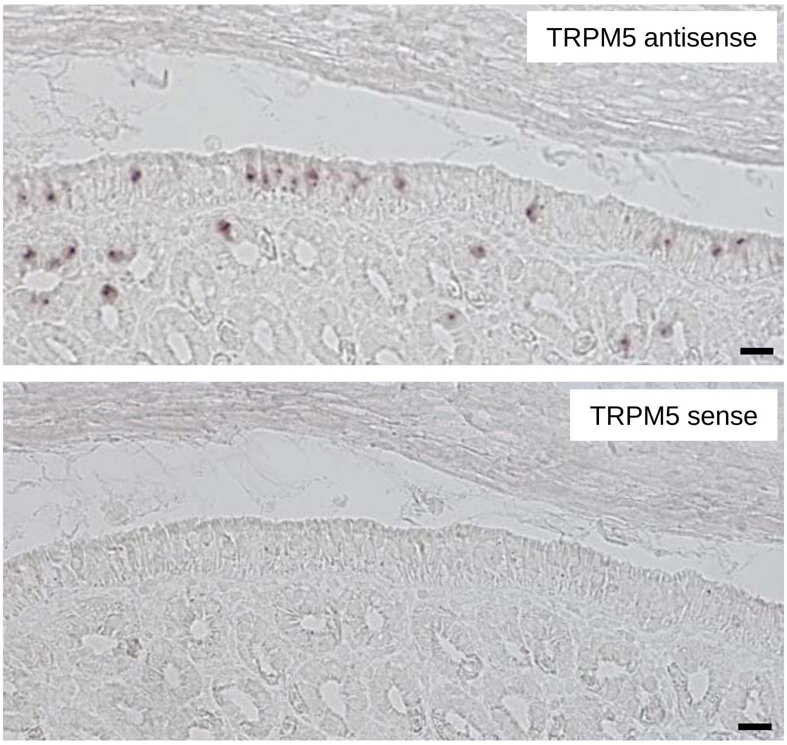

attached figure 6

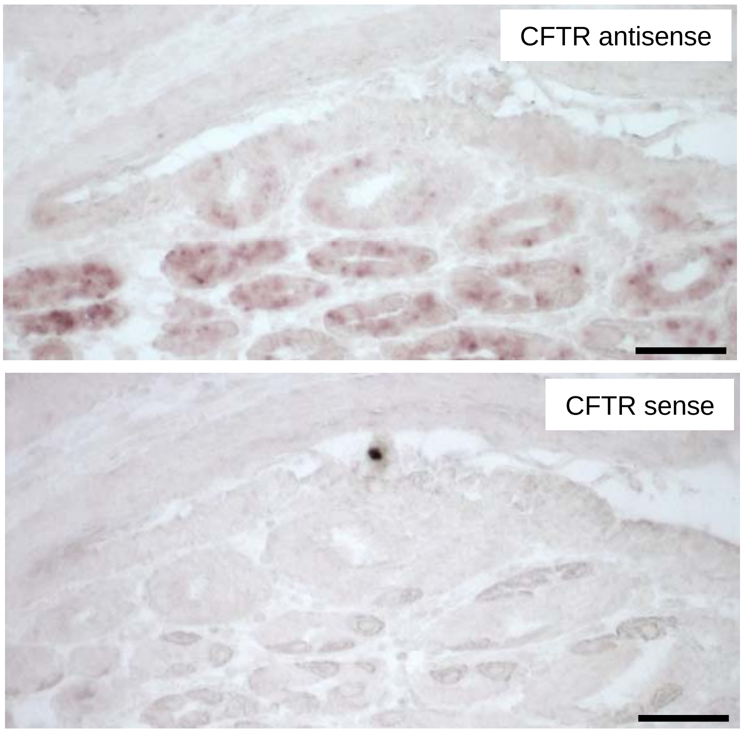

attached figure 7

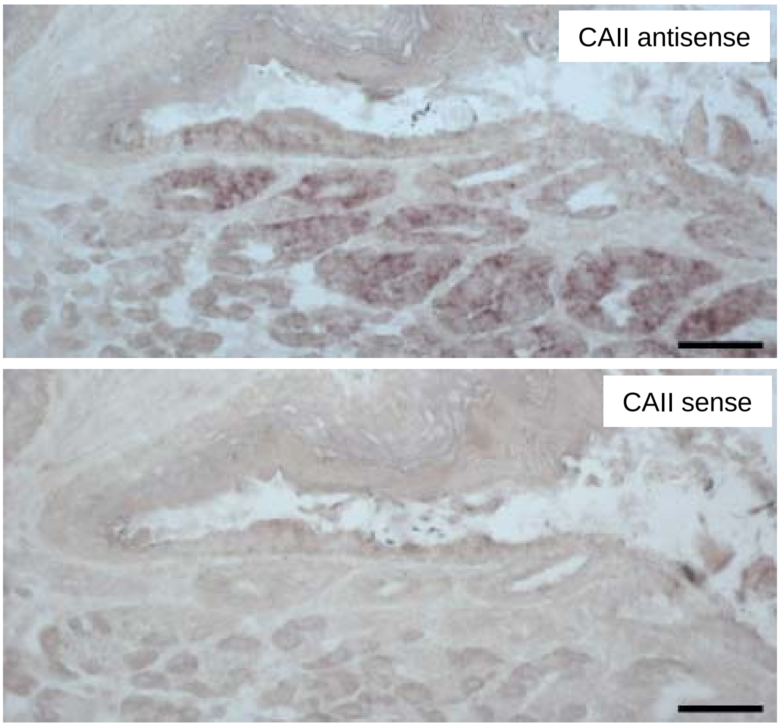

attached figure 8

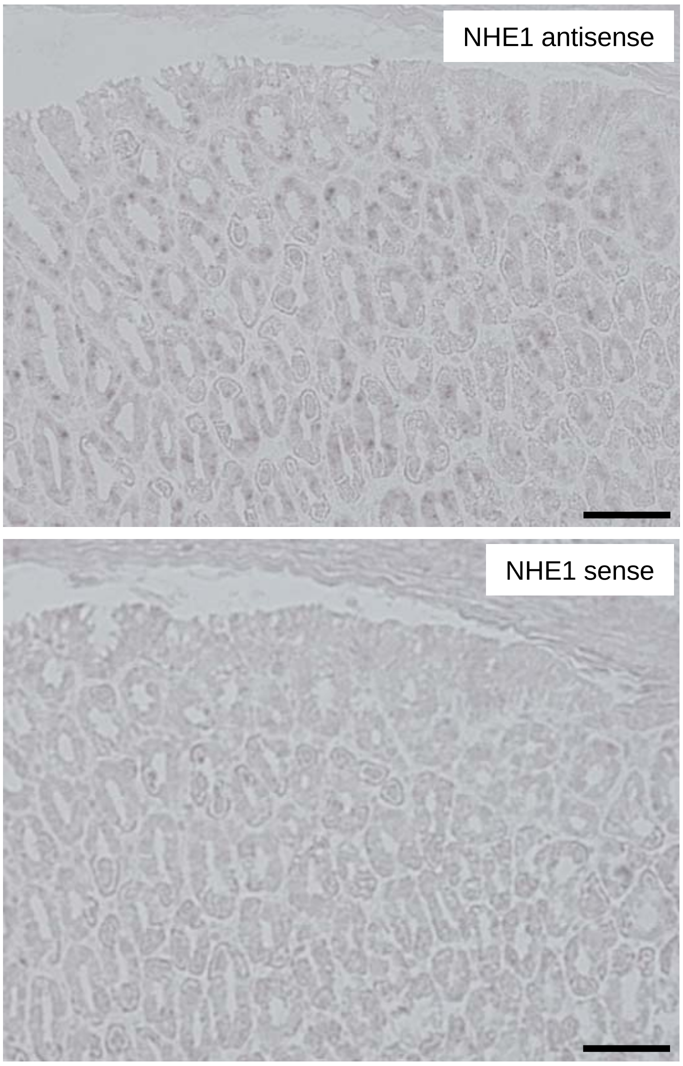

attached figure 9

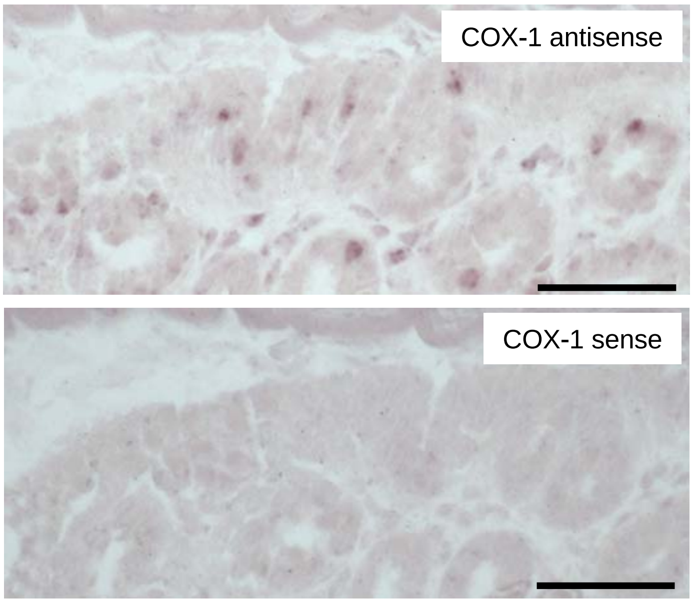

attached figure 10

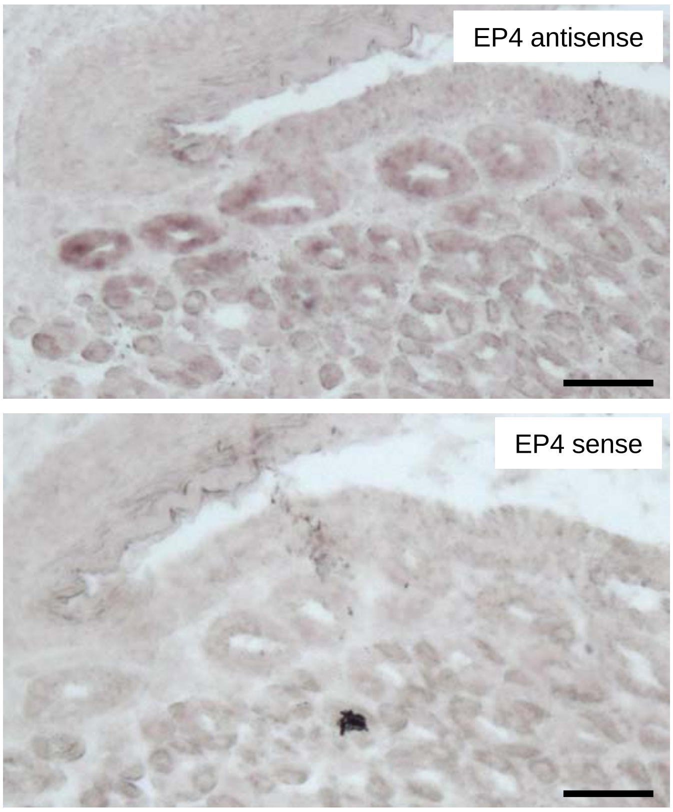

Supplement: Supplementary file 1 [file DataSheet1.PDF]
